# *LRIG1* gene copy number analysis by ddPCR and correlations to clinical factors in breast cancer

**DOI:** 10.1186/s12885-020-06919-w

**Published:** 2020-05-24

**Authors:** Mahmood Faraz, Andreas Tellström, Christina Edwinsdotter Ardnor, Kjell Grankvist, Lukasz Huminiecki, Björn Tavelin, Roger Henriksson, Håkan Hedman, Ingrid Ljuslinder

**Affiliations:** 1 Department of Radiation Sciences, Oncology, Umeå University, SE-90187 Umeå, Sweden; 2 Department of Medical Biosciences, Umeå University, SE-90187 Umeå, Sweden; 3 National Bioinformatics Infrastructure Sweden, SciLifeLab, Uppsala, Sweden; 4 Current address: Instytut Genetyki i Hodowli Zwierząt Polskiej Akademii Nauk, ul. Postępu 36A, 05-552 Jastrzębiec, Magdalenka Poland

**Keywords:** Breast cancer, LRIG1, Gene copy number, ddPCR, Prognosis

## Abstract

**Background:**

Leucine-rich repeats and immunoglobulin-like domains 1 (*LRIG1*) copy number alterations and unbalanced gene recombination events have been reported to occur in breast cancer. Importantly, *LRIG1* loss was recently shown to predict early and late relapse in stage I-II breast cancer.

**Methods:**

We developed droplet digital PCR (ddPCR) assays for the determination of relative *LRIG1* copy numbers and used these assays to analyze *LRIG1* in twelve healthy individuals, 34 breast tumor samples previously analyzed by fluorescence in situ hybridization (FISH), and 423 breast tumor cytosols.

**Results:**

Four of the *LRIG1*/reference gene assays were found to be precise and robust, showing copy number ratios close to 1 (mean, 0.984; standard deviation, +/− 0.031) among the healthy control population. The correlation between the ddPCR assays and previous FISH results was low, possibly because of the different normalization strategies used. One in 34 breast tumors (2.9%) showed an unbalanced *LRIG1* recombination event. *LRIG1* copy number ratios were associated with the breast cancer subtype, steroid receptor status, *ERBB2* status, tumor grade, and nodal status. Both *LRIG1* loss and gain were associated with unfavorable metastasis-free survival; however, they did not remain significant prognostic factors after adjustment for common risk factors in the Cox regression analysis. Furthermore, *LRIG1* loss was not significantly associated with survival in stage I and II cases.

**Conclusions:**

Although *LRIG1* gene aberrations may be important determinants of breast cancer biology, and prognostic markers, the results of this study do not verify an important role for *LRIG1* copy number analyses in predicting the risk of relapse in early-stage breast cancer.

## Background

Breast cancer is the most common cancer which threatens the health of women, with increasing incidence and mortality rates [1, 2]. According to gene expression profiles, breast cancer is classified into four major subtypes: luminal A, luminal B, ERBB2-enriched (also called HER2-enriched), and basal-like (also called triple-negative breast cancer – TNBC) [3]. The four subtypes differ significantly with regard to incidence, response to therapy, and prognosis [4, 5]. Even though the prognosis has improved in recent years, the risk of local recurrence remains at 10% [6], and the distal recurrence rate is almost 30% [7]. The most important risk factors for breast cancer outcome are tumor size, nodal involvement, tumor grade, ERBB2 status, proliferation index, and hormone receptor status [8]. However, there is a great need for new reliable factors that can discriminate between women with a high and low risk of early and late recurrence [9].

Leucine-rich repeats and immunoglobulin-like domains protein 1 (LRIG1) is a tumor suppressor that regulates various receptor tyrosine kinases, including ERBB2 and other epidermal growth factor receptor family members [10–12]. In breast cancer, the regulation of LRIG1 expression and its impact on tumor cell fate are complex. Indeed, LRIG1 mRNA expression might be an independent prognostic marker in different subtypes of breast cancer. For example, Krig et al. found a correlation between LRIG1 mRNA expression and relapse-free survival of ER+, LN-, HER2- breast cancer patients. So in estrogen receptor (ER)-positive breast cancer, LRIG1 seems to participate in a negative feedback loop wherein estrogen signaling upregulates *LRIG1* expression, which leads to the suppression of cancer cell proliferation [12]. In contrast, a feed-forward loop seems to dominate in ERBB2-positive breast cancer. Thus, whereas LRIG1 suppresses ERBB2 expression and the proliferation of ERBB2-positive breast cancer cells, ERBB2 itself downregulates LRIG1 levels in breast cancer cells, thereby canceling the tumor-suppressive function of LRIG1 [13]. Additionally, LRIG1 seems to play an important role in basal-like breast cancer. LRIG1 suppresses epithelial-to-mesenchymal transition and invasion of basal-like breast cancer cells; however, LRIG1 is downregulated by unknown mechanisms in the majority of basal-like tumors [14]. Thus, LRIG1 may be an influential determinant of all the major subtypes of breast cancer, including ER-positive, ERBB2-positive, and basal-like breast tumors.

LRIG1 expression is often downregulated in cancer cells, and high expression is associated with improved survival in many cancer types (reviewed in [15]). In ER-positive and lymph node-negative breast cancer, *LRIG1* mRNA expression is correlated with prolonged relapse-free survival [12], and in a series of mixed breast cancer cohorts, low expression of *LRIG1* was correlated with a shorter distant metastasis-free survival (MFS) and overall survival (OS) [16]. The *LRIG1* gene has shown both increased and decreased copy numbers in breast cancer. In our previous studies, in which fluorescence in situ hybridization (FISH) was used to determine gene copy numbers, *LRIG1* showed increased and decreased copy numbers in 34 and 3.5% of breast tumors, respectively [17, 18]. However, in a more recent study of stage I-II patients, which utilized a molecular inversion probe analysis platform, only 3.9% of the breast cancers showed an increased *LRIG1* copy number, whereas 8.9% showed losses [16]. The same study also indicated a common breakpoint in *LRIG1*; however, the frequency of this event was not determined. Thus, the frequencies of *LRIG1* gains, losses, and breaks in breast cancer remain controversial. Intriguingly, the study by Thompson et al. [16] has demonstrated that *LRIG1* loss predicts both early and late relapse in early-stage breast cancer. This finding is of potentially urgent clinical importance because markers for risk of late relapse in early-stage breast cancer are urgently needed.

We undertook the current study to establish a simple, precise, and sensitive droplet digital polymerase chain reaction (ddPCR) assay for the quantification of *LRIG1* gene copy numbers in cells and tissues, and we applied this assay to investigate the frequency of unbalanced *LRIG1* gene recombination events in breast cancer, determine the frequency of *LRIG1* gains and losses in a well-characterized breast cancer cohort, and validate, or refute, the previous claim that *LRIG1* loss can predict early and late relapses in breast cancer. We also performed exploratory analyses and investigated other possible associations between *LRIG1* copy numbers and various clinical parameters of interest.

## Methods

### Droplet digital PCR

Primers and probes for ddPCR for the reference genes (Table S1) and different genomic positions of *LRIG1* (Table S2) were purchased from Integrated DNA Technologies (Leuven, Belgium). For *ERBB2*, a ready-to-use ddPCR copy number variation assay was purchased from Bio-Rad Laboratories AB (Solna, Sweden; cat ≠ 10,031,240). The final concentrations of forward and reverse primers were 400 nM for *LRIG1* and the reference genes and 900 nM for *ERBB2*. The final concentrations of the probes were 200 nM for *LRIG1* and the reference genes and 250 nM for *ERBB2*. ddPCR supermix (no dUTP) (Bio-Rad, cat ≠ 1,863,024), Hind III restriction enzyme (Thermo Scientific, FastDigest, cat ≠ FD0505), and nuclease-free water were mixed with primer/probe sets of *LRIG1* or *ERBB2* and primer/probe sets for the reference gene. Droplets were generated using a QX200 droplet generator followed by PCR using a T100 thermal cycler (Bio-Rad) with PCR parameters of 37 °C for 5 min; 95 °C for 5 min; 40 cycles of 30 s at 95 °C and 1 min at 58 °C; followed by 98 °C for 10 min. After PCR amplification, to acquire these data, the plate was loaded into the QX200 droplet reader (Bio-Rad). The data were analyzed using QuantaSoft software (Bio-Rad, version 1.7.4.0917). To provide good quality and consistent data, the amplitude thresholds were set to 3500 for *LRIG1* or *ERBB2* and 3000 for *CYP1B*1 in the 1-D and 2-D plots. If the total number of events was less than 8000 counts, they were not included in the final analysis. In addition, data with a coefficient of variation (CV) greater than 10% in technical replicates were removed to obtain a more precise estimation of the ratios. Researchers were blinded to the clinical data of the patients at the time of performing the ddPCR and data analysis.

### Patients and tumor samples

The breast cancer cohort analyzed in the current study comprised 423 unselected women from the Northern Region in Sweden diagnosed with primary invasive breast carcinoma between 1987 and 1999. We used the frozen cytosol samples which were prepared for steroid receptor analysis as previously described [19]. We did not purify the DNA from samples because preliminary experiments showed that the crude cytosols worked as efficiently as the purified DNA as templates in the ddPCR assays. The receptor concentration was expressed in femtomoles of receptor per μg of DNA, and tumors with a value lower than 0.1 fmol ER or progesterone receptor (PR) per μg of DNA were considered to be receptor-negative; those with a value ≥0.1 fmol ER or PR per μg of DNA were considered to be receptor-positive [20]. The International Union Against Cancer guidelines (UICC-TNM) for tumor classification and staging were used. Details of the characteristics of the patients are presented in Table 1. Primary treatment was administered according to the guidelines of the North Swedish Breast Cancer Group. Patients with node-negative disease had a modified radical mastectomy or sector resection, and the patients who underwent sector resection were treated with postoperative radiation therapy. Moreover, 60 patients received adjuvant chemotherapy, and 145 patients received adjuvant endocrine treatment, with most cases receiving tamoxifen daily for 2 to 5 years. Patients with node-positive disease were treated with modified radical mastectomy, axillary dissection, and postoperative radiation therapy. The number of patients for whom data were available varied among the different prognostic factors studied depending on the clinical routines at the time of collection of the respective sample. Information on the histopathologic grade was available in 363 cases. The median age at diagnosis was 60 years. The last follow-up dates for OS and MFS were June 30, 2017 and December 31, 2013, respectively. Clinical information including primary stage, adjuvant therapy, time and type of relapses, and survival, was obtained from national registries and from patient records when available. Because the cohort was more than 25 years old, reliable treatment data for patients with primary metastasized disease and/or recurrence could not be obtained. The patients who were diagnosed with stage IV disease less than 6 months after their original breast cancer diagnosis were classified as primary stage IV cases in our analysis of recurrence risk. Among the 154 patients who had died from breast cancer at the last follow-up date, the date of recurrence was obtained in 96 of 154 patients and was used when analyzing time to recurrence. MFS and OS were calculated as the time from diagnosis to the date of first recurrence or death. The follow-up times for patients without documented recurrences or death were calculated as the time from diagnosis until the last clinical examination (last follow-up date, December 31, 2013).

**Table 1:** *LRIG1* copy number ratios and clinicopathological characteristics of the breast cancer cohort

Characteristic	Loss^**a**^	Normal^**a**^	Gain^**a**^	***P***-value*
**Number of patients (*N*** **= 423**	77 (18.2%)	293 (69.3%)	53 (12.5%)	
**Age at diagnosis (years; mean ± SD)**	55.7 ± 13.3	60.1 ± 11.54	62.5 ± 13.42	
Age ≤ 60 (*N* = 211)	46 (21.8%)	142 (67.3%)	23 (10.9%)	0.125
Age > 60 (*N* = 212)	31 (14.6%)	151 (71.2%)	30 (14.2%)	
Steroid receptor status				**< 0.001**
Negative (*N* = 117)	39 (33.3%)	65 (55.6%)	13 (11.1%)	
Positive (*N* = 306)	38 (12.4%)	228 (74.5%)	40 (13.1%)	
***ERBB2*status**				**0.002**
Negative (*N* = 336)	50 (14.9%)	245 (72.9%)	41 (12.2%)	
Positive (*N* = 87)	27 (31.0%)	48 (55.2%)	12 (13.8%)	
Tumor subtype				**< 0.001**
*ERBB2*+, ER/PR- (*N* = 45)	16 (35.6%)	23 (51.1%)	6 (13.3%)	
*ERBB2*+, ER/PR+ (N = 42)	11 (26.2%)	25 (59.5%)	6 (14.3%)	
*ERBB2*-, ER/PR+ (*N* = 264)	27 (10.2%)	203 (76.9%)	34 (12.9%)	
*ERBB2*-, ER/PR- (*N* = 72)	23 (31.9%)	42 (58.3%)	7 (9.7%)	
**Disease stage (*N*** **= 278; missing data = 145)**				0.151
I (*N* = 109)	18 (16.5%)	84 (77.1%)	7 (6.4%)	
II (*N* = 131)	28 (21.4%)	85 (64.9%)	18 (13.7%)	
III (N = 7)	1 (14.3%)	5 (71.4%)	1 (14.3%)	
IV (*N* = 31)	6 (19.4%)	17 (54.8%)	8 (25.8%)	
**Tumor grade (*N*** **= 363, missing data = 60)**				**0.004**
I (N = 31)	1 (3.2%)	25 (80.6%)	5 (16.1%)	
II (*N* = 137)	15 (10.9%)	104 (75.9%)	18 (13.1%)	
III (*N* = 195)	50 (25.6%)	120 (61.5%)	25 (12.8%)	
**Tumor size (*N*** **= 329, missing data = 94)**				0.158
Size ≤20 mm (*N* = 154)	29 (18.8%)	111 (72.1%)	14 (9.1%)	
Size > 20 mm (*N* = 175)	37 (21.1%)	111 (63.4%)	27 (15.4%)	
**Nodal status (*N*** **= 360, missing data = 63)**				**0.001**
Negative (*N* = 279)	43 (15.4%)	207 (74.2%)	29 (10.4%)	
Positive (*N* = 81)	24 (29.6%)	43 (53.1%)	14 (17.3%)	
Tumor types				**0.041**
Ductal (*N* = 354)	66 (18.6%)	237 (66.9%)	51 (14.4%)	
Lobular (N = 35)	5 (14.3%)	30 (85.7%)	0 (0.0%)	
Others (*N* = 34)	6 (17.6%)	26 (76.5%)	2 (5.9%)	

^a^*LRIG1*/*CYP1B1* ratio < 0.85, loss; 0.85–1.15, normal; > 1.15, gain

*The overall *P* values are from comparisons between all *LRIG1* loss, *LRIG1* normal and *LRIG1* gain groups. Significance was calculated by the 2-sided Fisher’s exact test

### Statistical analyses

All statistical analyses were performed using SPSS software, version 24 (IBM Corporation, Armonk, NY, USA). The Spearman correlation coefficient was used to evaluate the correlation between the *LRIG1* copy number and *ERBB2* copy number ratios. The Kruskal-Wallis test was conducted to evaluate whether the distribution of *LRIG1* copy number ratios was the same among different subtypes. Fisher’s exact test (2-sided) was used to investigate the relationships between *LRIG1* loss or gain with all other variables used in the cohort (Table 1). The survival analysis data were presented with Kaplan-Meier survival curves and evaluated by the log-rank (Mantel-cox) test. Cox regression analysis was also conducted for both OS and MFS, including *LRIG1* loss or gain together with other prognostic factors. In all statistical analyses, the significance level was set to 0.05.

## Results

### Identification of reference genes and design and validation of ddPCR assays

As candidate genomic reference loci, we chose six loci with a low copy number variance in breast cancer. Thus, we excluded chromosome arms and regions that were previously shown to display frequent copy number alterations in early-stage breast cancer [21], i.e., chromosomes 1q, 8, 11, 16, 17, and 20, as well as all other regions that showed gains or losses in ≥10% of any of the major breast cancer subtypes. Among the remaining chromosomal regions, we attempted to manually identify one or more genes per chromosome arm. However, we failed to identify suitable genes in the low-variance parts of chromosomes 4, 5, 6, 10p, 12p, 13p, 14, 15p, 18p, 21p, 22, or Xp. In total, 23 genes on 17 different chromosome arms were identified and chosen for further evaluation (Table S3). The copy number variance among these 23 genes was analyzed in the cancer genome atlas (TCGA) breast cancer data set, revealing a frequency of copy number changes in the TCGA cohort between 0.94 and 4.1% (Table S3). *LRIG2* was excluded as a reference gene in the present study due to an apparent risk that its copy number might not be independent of the studied gene, *LRIG1*. Thereafter, ddPCR assays for the six reference genes that showed the lowest frequency of copy number variation in the TCGA data set and, simultaneously, were located on different chromosomal arms, were designed (Table S1). Additionally, ddPCR assays for six loci along the *LRIG1* gene were designed (Table S2). The performance of all twelve ddPCR assays was good, with PCR amplification efficiencies > 94% (95% confidence intervals [CIs] for all assays were within 0.93 < 1.02) and good linearity (r^2^ = 1.00 for all assays) when synthetic DNA was used as the PCR template. Next, six different *LRIG1*/reference gene duplex assays were used to analyze the chromosomal DNA from twelve healthy individuals (Table S4). Four of the six assay pairs, i.e., *LRIG1–9*/*GJB2*, *LRIG1–11*/*CHUK*, *LRIG1–7*/*CYP1B1*, and *LRIG1–12*/*NR5A1*, showed ratios that were very close to 1 in all samples (mean ratios, ± standard deviations [SD]: 0.997, ± 0.050; 0.991, ± 0.029; 0,979, ± 0.041; and 0.968, ± 0.030, respectively). When these four assays were combined and used to determine the *LRIG1* copy number among the twelve healthy individuals, the apparent mean copy number ratios were, on average, 0.984 (SD, ± 0.031; 95% CI, 0.966–1.002).

### *LRIG1* and *ERBB2* copy number variations in breast cancer tumors

**Fig. 1:**
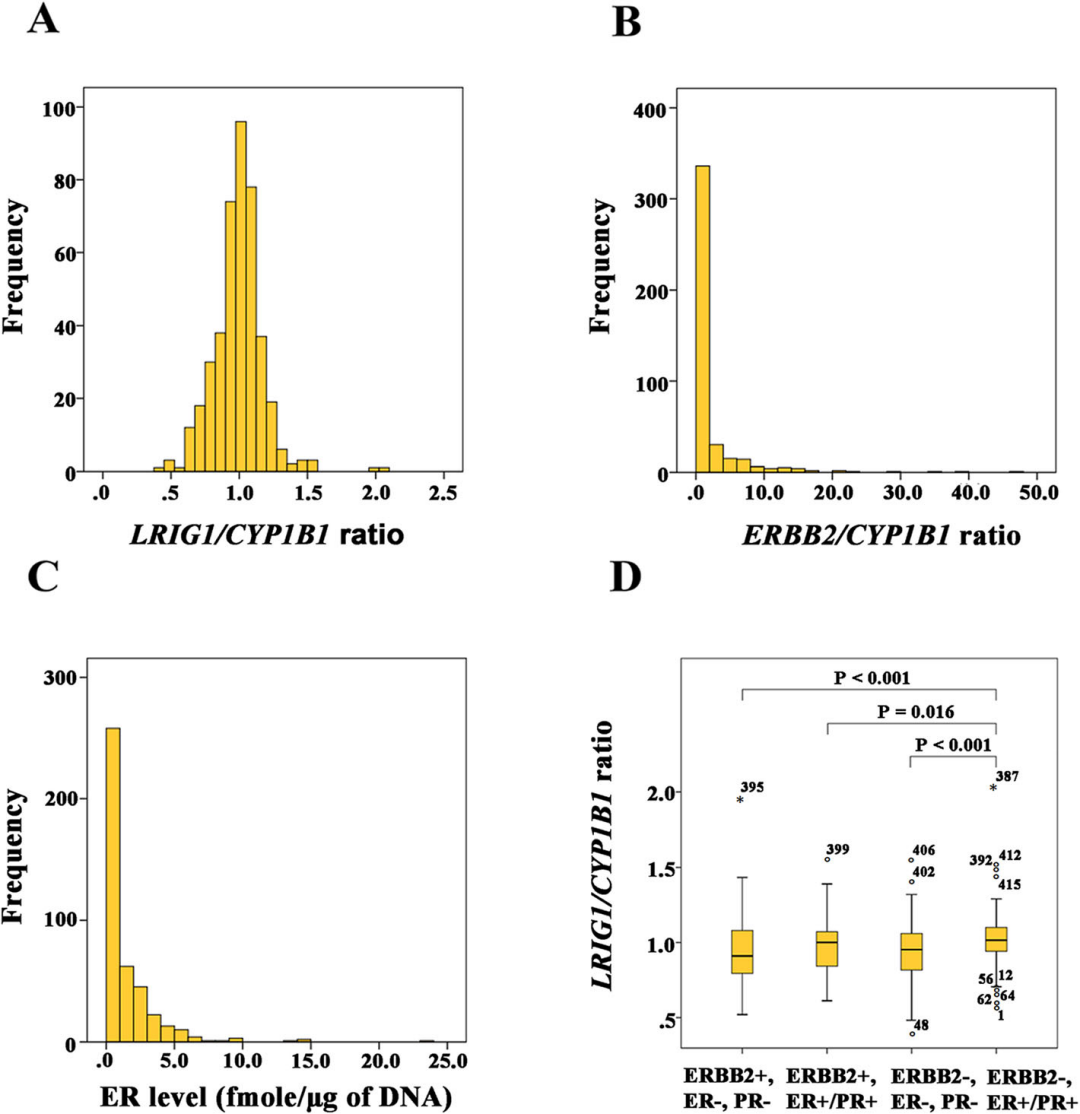
Frequency distributions of *LRIG1* and *ERBB2* copy number ratios and ER levels and relationships between *LRIG1* copy number ratios and breast cancer subtypes among 423 breast cancer cases. **a** Frequency distributions of *LRIG1*/*CYP1B1* ratios determined by ddPCR. **b** Frequency distributions of *ERBB2*/*CYP1B1* ratios determined by ddPCR (**c**) Frequency distributions of ER levels retrieved from clinical records. **d** Box plots of *LRIG1*/*CYP1B1* ratios for each tumor subtype

The four *LRIG1*/reference gene ddPCR assay pairs that had shown the ratios closest to 1 among the samples from the healthy individuals were thereafter used to analyze DNA from 34 breast cancer tumors that had been analyzed for *LRIG1* copy number variations by FISH in a previous study [18]. The major clinical characteristics of these patients are presented in Table S5. To detect unbalanced gene recombination events, we analyzed the SD among the ratios for the four assays that were distributed along the *LRIG1* gene. One sample showed an aberrant SD that was greater than 0.1 (SD, ± 0.431), thus representing a probable unbalanced gene recombination event. Based on this finding, we concluded that 2.9% (1/34) of the breast tumors in this series had undergone an unbalanced *LRIG1* gene recombination event. We used the same cut-offs as were used by us in the paper by Thompson et al., (2014); that is, the definition of loss was an LRIG1-ratio <  0.85 and of gain a ratio > 1.15, that is delta +/− 0.15 around 1.00. Using these thresholds, 11.8% (4/34) of the tumors showed *LRIG1* loss and 2.9% (1/34) showed *LRIG1* gain. Intriguingly, only one in seven tumors that had previously shown *LRIG1* gain by FISH also showed an *LRIG1* gain by the ddPCR assay. In fact, there was a poor correlation between the *LRIG1* copy number ratios determined by ddPCR and the *LRIG1* copy numbers previously determined by FISH (linear regression, y = 1.004 + 0.100x, r^2^ = 0.009; Fig. S1). Finally, we analyzed the *LRIG1*/*CYP1B1* ratio and *ERBB2*/*CYP1B1* ratio in 423 breast cancer tumor cytosols. Here, only a single reference gene, *CYP1B1*, was used, to reduce the number of ddPCR runs. Figure 1a and b show the distribution of *LRIG1*/*CYP1B1* and *ERBB2*/*CYP1B1* copy number ratios, respectively, among the 423 tumors. Using cut-offs < 0.85 for *LRIG1* loss and > 1.15 for *LRIG1* gain, 18.2% of the tumors showed loss and 12.5% showed gain (Table 1). The samples with *ERBB2*/*CYP1B1* ratios ≥2 were defined as *ERBB2*-positive tumors (according to the guideline recommendations of the American Society of Clinical Oncology/College of American Pathologists), which corresponded to 20.6% of all tumors. Using continuous data, *LRIG1* and *ERBB2* copy number ratios were correlated (*P* = 0.016, Spearman’s ρ correlation coefficient = 0.117). Nevertheless, *LRIG1* loss was more common among the *ERBB2*-positive (31%) than among the *ERBB2*-negative (14.9%) tumors (*P* = 0.001, Fisher’s exact test, 2-sided). The frequency of *LRIG1* gains did not differ between the *ERBB2*-positive and *ERBB2*-negative tumors (*P* = 0.323, Fisher’s exact test, 2-sided).

We also investigated the effects of minor changes of the cut-off levels. New cut-offs were tested with delta from 0.15 up to 0.25 with step 0.01. When these alternative cut-off definitions were tested in the full model, together with the other prognostic factors, each level of LRIG-ratio was found to be non-significant. This means that the definition of loss and gain used in the manuscript was stable and not dependent on minor changes in the predefined cut-offs.

### Associations between *LRIG1* losses or gains and various clinical parameters

Figure 1c shows the distribution of ER levels in the cohort. The median and mean values of ER were 0.6 fmol/μg of DNA and 1.4 fmol/μg of DNA, respectively (range from 0.0 to 23.0 fmol/μg of DNA). The median and mean values of PR were 0.4 fmol/μg of DNA and 1.4 fmol/μg of DNA, respectively (range from 0.0 to 22.0 fmol/μg of DNA). *LRIG1* loss was more common among steroid receptor-negative (33.3%) than among steroid receptor-positive (12.4%) tumors (*P* <  0.001, Fisher’s exact test, 2-sided) (Table 1). The frequency of *LRIG1* gain did not differ between steroid receptor-negative and steroid receptor-positive tumors (*P* = 0.722, Fisher’s exact test, 2-sided). We defined four breast cancer subtypes in our study based on the data for *ERBB2* copy numbers and ER and PR receptor statuses: *ERBB2*+, ER/PR- (i.e., *ERBB2*+, ER-, PR-); *ERBB2*+, ER/PR+ (i.e., *ERBB2*+, ER+, PR-; *ERBB2*+, ER-, PR+; or *ERBB2*+, ER+, PR+); *ERBB2*-, ER/PR+ (i.e., *ERBB2*-, ER+, PR-; *ERBB2*-, ER-, PR+; or *ERBB2*-, ER+, PR+); and *ERBB2*-, ER/PR- (i.e., *ERBB2*-, ER-, PR-). Figure 1d shows the *LRIG1* copy number ratios among the breast cancer subtypes. *LRIG1* copy number ratios were different among the groups (*P* <  0.001, Kruskal-Wallis test). In a pairwise comparison, *LRIG1* loss was less common among the *ERBB2*-, ER/PR+ tumors than the other subtypes (*P* = 0.016, Fisher’s exact test, 2-sided). We defined disease stage from I to IV based on the TNM staging system. The TNM data for 145 patients were missing. There were only seven stage III patients, among whom only one patient had a loss and another had a gain. The frequencies of *LRIG1* loss did not differ among various disease stages (Fisher’s exact test); however, *LRIG1* gain was more common in stage IV than in stage I (*P* = 0.004, Fisher’s exact test, 2-sided). Tumor grade data were available for 363 patients. Among those tumors, *LRIG1* loss was more common among grade 3 tumors than among grade 1 tumors and was more common among grade 3 tumors than among grade 2 tumors; however, there was no difference between grade 1 and grade 2 tumors (*P* = 0.004, *P* = 0.001, and *P* = 0.305, respectively, Fisher’s exact test, 2-sided). *LRIG1* gain was equally common among the different tumor grades (Fisher’s exact test). *LRIG1* copy number ratios were not correlated with tumor size. Both *LRIG1* loss and gain were significantly correlated with nodal status (*P* = 0.002, and *P* = 0.035, respectively, Fisher’s exact test, 2-sided). Node-positive tumors had more *LRIG1* losses or gains than node-negative tumors. The frequencies of *LRIG1* losses differed among ductal, lobular, and “others” tumor types (*P* = 0.041, Fisher’s exact test). Among the tumors with lobular cancer, no *LRIG1* gain was found (0/35).

### Patient survival analyses

**Fig. 2:**
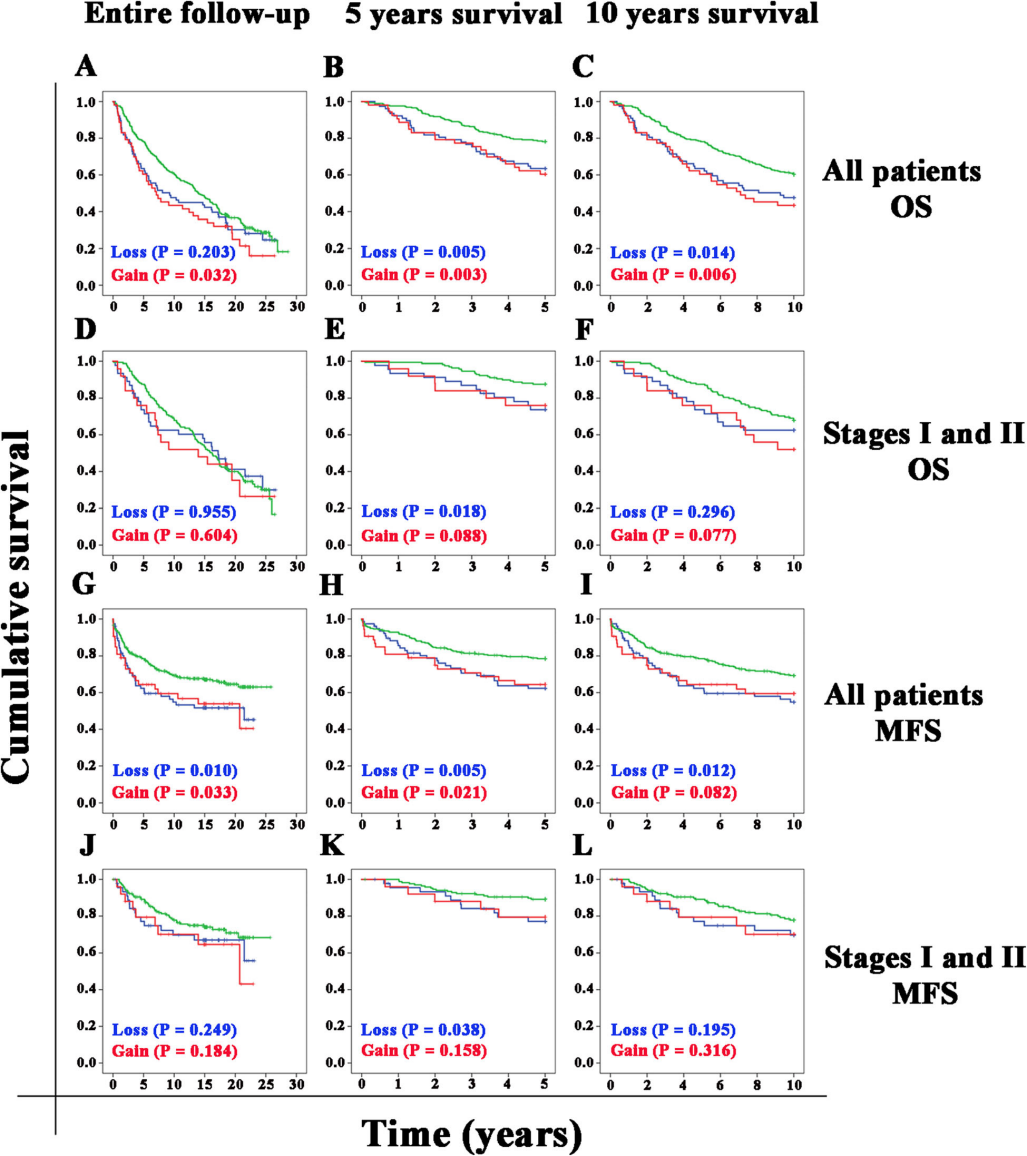
Kaplan-Meier curves for OS and MFS according to *LRIG1* status. Kaplan-Meier analyses were performed for OS (A-F) or MFS (G-L) for 423 breast cancer patients according to *LRIG1* status (^**___**^ normal *LRIG1,*^**___**^*LRIG1* loss, ^**___**^*LRIG1* gain). Analyses are presented for the entire follow-up time (**a**, **d**, **g**, and **j**), five-year survival (**b**, **e**, **h**, and **k**), or ten-year survival (**c**, **f**, **i**, and **l**). Statistical significance was calculated using the log-rank test and is indicated in each graph

First, we confirmed the associations between known prognostic factors and patient MFS in our cohort by applying the Mantel-Cox log-rank tests (Fig. S2). Steroid receptor-negative patients had a worse MFS than steroid receptor-positive patients (*P* <  0.001, Fig. S2A). *ERBB2*-amplification was strongly correlated with a worse MFS (*P* <  0.001, Fig. S2B). Among our four defined breast cancer subtypes, the *ERBB2*-, ER/PR+ subtype showed the best MFS, whereas the *ERBB2*+, ER/PR- subtype had the worst prognosis (Fig. S2C). There were significant differences in MFS between the *ERBB2*-, ER/PR+ subtype and all other subtypes (*P* = 0.002) and between the *ERBB2*+, ER/PR- and *ERBB2*-, ER/PR- subtypes (*P* = 0.048) (*P* <  0.001). Tumor grade stratified patients into three different prognostic groups, among which patients with a higher grade had a worse MFS (*P* = 0.014, Fig. S2D). Similarly, tumor size stratified the patients into three different prognostic groups for MFS (T1 vs T2: *P* = 0.039; T1 vs T3: *P* <  0.001; T2 vs T3: *P* = 0.002, Fig. S2E). Regarding nodal status, both N1 and N2 patients had a significantly worse MFS than node-negative (N0) patients (*P* <  0.001 and *P* = 0.001, respectively, Fig. S2F). Patients with distant metastases at diagnosis (M1) showed a significantly worse survival than patients without distant metastases at diagnosis (M0) (*P* <  0.001, Fig. S2G). Metastasis and death due to breast cancer were defined as events in the metastasis-free survival analyses. All comparisons among the disease stages were significant (*P* ≤ 0.001). Patients with higher stages of disease had a worse MFS than patients with lower stages (Fig. S2H). We used the Mantel-Cox log-rank test to calculate the significance level of differences between OS or MFS distributions for the different *LRIG1* copy number categories (loss, normal, or gain) for the whole cohort or early-stage breast cancer (stages I and II), for the entire study period, and for 5 years and 10 years (Fig. 2). The overall survival analysis for all patients demonstrated that patients with *LRIG1* gain, but not *LRIG1* loss, had a worse prognosis than patients with a normal *LRIG1* copy number (Fig. 2a). However, for 5-year survival (Fig. 2b) or 10-year survival (Fig. 2c), patients with either *LRIG1* loss or *LRIG1* gain had a significantly worse OS than patients with a normal *LRIG1* copy number. The overall survival analysis for early-stage patients revealed no significant differences between patients with *LRIG1* loss or gain and patients with a normal *LRIG1* copy number (Fig. 2d). However, for 5-year OS (Fig. 2e), but not for 10-year OS (Fig. 2f), patients with *LRIG1* loss had a significantly worse OS than patients with a normal *LRIG1* copy number (Fig. 2e and f). In the entire cohort, both patients with *LRIG1* loss and *LRIG1* gain had a significantly worse MFS than patients with a normal *LRIG1* copy number (Fig. 2g). This pattern was also observed for 5- and 10-year MFS (Fig. 2h and i). However, for stage I and II patients, only patients with *LRIG1* loss in the 5-year MFS analysis showed a significant difference compared with the patients with a normal *LRIG1* copy number (Fig. 2j-l). For the early-stage patients who relapsed, the median time to relapse was 43.4 months for patients with *LRIG1* loss and 68.5 months for patients with a normal *LRIG1* copy number. In our primary Cox regression model (Table 2), we included all the variables that significantly affected OS or MFS in our univariate analyses, i.e., tumor subtype, tumor grade, tumor size, nodal status, and patient age at diagnosis and *LRIG1* loss or gain. In this model, tumor subtypes and nodal status were independent prognostic factors both for OS and MFS, whereas tumor size and age at diagnosis were independent prognostic factors for OS only. However, neither *LRIG1* loss nor *LRIG1* gain showed a significant independent association with patient OS or MFS. Moreover, we did statistical analyses using the cause-specific breast cancer survival estimates together with the metastasis-free survival, but the results were very similar.

**Table 2:** Cox regression analysis of *LRIG1* loss, normal, and gain adjusted for all variables in all patients

Tumor characteristic	Overall survival		Metastasis-free survival	
	Hazard ratio (95% CI)	***P***-value	Hazard ratio (95% CI)	***P***-value
**Age at diagnosis**				
≤ 50 years	Reference			
> 50 years	2.633 (1.802–3.848)	< 0.001	1.306 (0.828–2.060)	0.251
**Tumor subtype**				
*ERBB*2-, ER/PR+	Reference			
*ERBB2*+, ER/PR-	1.558 (1.003–2.420)	0.049	1.915 (1.077–3.405)	0.027
*ERBB2*+, ER/PR+	1.563 (0.974–2.507)	0.064	1.983 (1.101–3.573)	0.023
*ERBB2*-, ER/PR-	1.384 (0.939–2.038)	0.1	1.583 (0.936–2.679)	0.087
**Grade**				
Low (1 or 2)	Reference			
High (3)	1.0 (1.0–1.0)	0.710	1.583 (0.936–2.679)	0.087
**Tumor size**				
≤ 20 mm	Reference			
> 20 mm	1.427 (1.067–1.908)	0.017	1.151 (0.751–1.762)	0.519
**Nodal status**				
Negative	Reference			
Positive	2.592 (1.840–3.651)	< 0.001	3.435 (2.206–5.347)	< 0.001
***LRIG1*copy number**				
Normal	Reference			
Loss	0.964 (0.668–1.393)	0.847	1.115 (0.690–1.803)	0.657
Gain	0.837 (0.550–1.274)	0.407	1.041 (0.585–1.852)	0.892

## Discussion

The identification of prognostic markers for risk of relapse in breast cancer is of major importance, and loss of *LRIG1* has indeed been shown to be a strong candidate marker for the risk of relapse in a stage I-II American breast cancer cohort [16]. To critically evaluate *LRIG1* loss as a prognostic marker in other breast cancer cohorts, we devised a precise and robust ddPCR method to assess *LRIG1* copy number ratios and applied this method to analyze *LRIG1* copy numbers in a healthy control population and a breast cancer cohort from northern Sweden. Among 423 stage I-IV breast cancer cases with a long follow-up period (20 years), we investigated possible associations between *LRIG1* copy number and patient survival and various clinical factors. Thereby, we could confirm some and refute other previously published observations regarding *LRIG1* copy number associations in breast cancer.

In accordance with previous studies showing that LRIG1 expression is higher in ERα-positive than in ERα-negative tumors [12] and decreased in ERBB2-positive compared with ERBB2-negative tumors [13], we found that *LRIG1* loss was more common among steroid receptor-negative tumors and *ERBB2*-positive tumors than among steroid receptor-positive and *ERBB2*-negative tumors, respectively. In contrast to the results obtained previously [16], however, we found that *LRIG1* loss was significantly correlated with tumor grade and nodal status. Thus, in the present study, the frequency of *LRIG1* loss seemed to increase together with increasing aggressiveness of the tumor. Moreover, we could confirm that *LRIG1* copy numbers were associated with the breast cancer tumor subtype, although the tumor subtype criteria used in the current study, applied to relatively old clinical material, were slightly different from the more modern criteria used by Thompson et al. [16].

A genomic breakpoint has been speculated [18] and shown [16] to be present in *LRIG1* in breast cancer; however, the prevalence of this genomic alteration has not been determined previously. Here, we could show that one in 34 breast tumors (2.9%) in our cohort appeared to display an unbalanced *LRIG1* recombination event. Thus, the frequency of unbalanced *LRIG1* recombination events does not seem to be very high in breast cancer, although it will be interesting to analyze larger breast cancer data sets, such as the TCGA data sets, to acquire more reliable estimates of the frequency and to resolve whether specific breast cancer subtypes are predominantly associated with this event.

In the present study, 12.5% of the tumors displayed *LRIG1* gains, contrasting with our previous FISH results [17, 18] showing that 39% of breast tumors displayed *LRIG1* gains. In an effort to clarify this discordance, we applied our new ddPCR method to analyze 34 tumors that had previously been analyzed by FISH. The results obtained with ddPCR showed a striking discordance with the previous FISH results. In fact, there was almost no correlation between the results of the two methods. This discordance could not be explained by any difference between the samples analyzed because the ddPCR and FISH analyses were performed on the same material, i.e., the same preparation of cell nuclei from each tumor. In the present ddPCR study, we used a reference gene on another chromosome to normalize the *LRIG1* copy number according to the cell number and tumor ploidy, whereas in the previous study, the *LRIG1* FISH signals were only normalized to the number of cells, i.e., the number of cell nuclei. It is possible that the lack of agreement between the ddPCR and FISH results might originate from the different normalization strategies used. Hence, we propose that the increased *LRIG1* copy numbers previously observed by FISH in most cases may reflect a general polyploidy of the tumor cells rather than specific increases in the *LRIG1* gene dosage.

Although the overall prognosis of breast cancer has recently improved [22], many patients still experience recurrence. Therefore, there is a great need for new and reliable tools to predict outcomes and to select the appropriate therapy. Regarding the prognostic value of *LRIG1* copy number alterations, both *LRIG1* loss and *LRIG1* gain were associated with an unfavorable MSF in this study, both for the whole follow-up time and for the 5-year and 10-year survival studies. Thus, *LRIG1* status predicted both early and late relapses in our cohort. However, in a multivariate Cox regression analysis, neither *LRIG1* loss nor *LRIG1* gain was an independent prognostic factor after adjustment for the tumor subtype, tumor grade, *LRIG1* copy number status, tumor size, nodal status, and age at diagnosis. Only tumor subtype and nodal status were found to be independent prognostic factors in this analysis. Moreover, among the stage I and II cases, neither *LRIG1* loss nor *LRIG1* gain was significantly associated with patient survival for the whole study period or the 10-year follow-up. Taken together, these analyses suggest that the observed correlations between *LRIG1* status and MFS in the present cohort were probably mostly due to associations between *LRIG1* status and tumor subtype and nodal status. These results contrast with our previous demonstration that *LRIG1* loss predicts early and late relapses of early-stage breast cancer [16]. The reason for the discordance between our two studies is not known. However, possible explanations include the differences between the patient cohorts analyzed and the analytical methods used. The current cohort comprised 423 patients in total, of whom only 240 were stage I-II, whereas the American cohort comprised 972 patients of stage I-II. In the current cohort, ethnicity was not recorded; however, it is likely that the ethnic compositions of the cohorts were different, which could be highly relevant because black and Hispanic populations are known to have a higher proportion of basal-like and ERBB2-positive tumors than non-Hispanic white populations, and indeed, the frequency of *LRIG1* loss differed among these groups in the American cohort [16]. Moreover, another factor with a potential major impact on patient outcome concerns the treatment differences between the cohorts. Regrettably, complete treatment records were not available for the patients in the current study. Another shortcoming of our study was that our clinical material did not comprise mRNA, and therefore LRIG1 mRNA expression analysis could not be performed to clarify its potential role as a prognostic factor in breast cancer. Accordingly, neither was the correlation between LRIG1 gene copy number and *LRIG1* expression analyzed in this study. It will be important to further assess these associations in larger breast cancer data sets, such as those available from TCGA, the International Cancer Genome Consortium, and the Molecular Taxonomy of Breast Cancer International Consortium.

## Conclusions

By using a novel ddPCR-based *LRIG1* copy number assay, we have shown that *LRIG1* loss is associated with nodal status and other clinical parameters; however, we could not verify *LRIG1* loss as a robust independent predictor of the risk of relapse in breast cancer. Thus, *LRIG1* gene aberrations may be important biological determinants of various aspects of breast cancer biology, and considered as prognostic markers, but the role of this gene as an independent predictor of relapse in breast cancer appears uncertain.

## Supplementary information

**Additional file 1: Fig. S1.** Lack of a correlation between the ddPCR and FISH results. Dot plot showing *LRIG1* copy numbers determined for 34 breast tumors using ddPCR (current study) and using FISH (Ljuslinder et al., 2009). The linear regression line (y = 1.004 + 0.100x, r^2^ = 0.009) is presented as a broken blue line.

**Additional file 2: Fig. S2.** Kaplan-Meier curves for MFS according to known risk factors. Kaplan-Meier analyses were performed for 423 breast cancer patients according to ER status (A), ERBB2 status (B), breast cancer subtype (C), tumor grade (D), tumor size (E), nodal status (F), distant metastasis (G), and disease stage (H). Statistical significance was calculated using the log-rank test and is indicated in each graph.

**Additional file 3.**

## Data Availability

The data sets are available from the corresponding author on reasonable request.

## Abbreviations

CI: Confidence interval
CV: Coefficient of variation
ddPCR: Droplet digital PCR
ER: Estrogen receptor
FISH: Fluorescence in situ hybridization
LRIG1: Leucine-rich repeats and immunoglobulin-like domains 1
MFS: Metastasis-free survival
OS: Overall survival
PCR: Polymerase chain reaction
PR: Progesterone receptor
SD: Standard deviation
TCGA: The cancer genome atlas

## References

[1] Ferlay J, Shin HR, Bray F, Forman D, Mathers C, Parkin DM (2010). Estimates of worldwide burden of cancer in 2008: GLOBOCAN 2008. Int J Cancer.

[2] DeSantis C, Ma J, Bryan L, Jemal A (2014). Breast cancer statistics, 2013. CA Cancer J Clin.

[3] Perou CM, Sorlie T, Eisen MB, van de Rijn M, Jeffrey SS, Rees CA, Pollack JR, Ross DT, Johnsen H, Akslen LA (2000). Molecular portraits of human breast tumours. Nature..

[4] Sorlie T, Perou CM, Tibshirani R, Aas T, Geisler S, Johnsen H, Hastie T, Eisen MB, van de Rijn M, Jeffrey SS (2001). Gene expression patterns of breast carcinomas distinguish tumor subclasses with clinical implications. Proc Natl Acad Sci U S A.

[5] Lehmann BD, Bauer JA, Chen X, Sanders ME, Chakravarthy AB, Shyr Y, Pietenpol JA (2011). Identification of human triple-negative breast cancer subtypes and preclinical models for selection of targeted therapies. J Clin Invest.

[6] Lowery AJ, Kell MR, Glynn RW, Kerin MJ, Sweeney KJ (2012). Locoregional recurrence after breast cancer surgery: a systematic review by receptor phenotype. Breast Cancer Res Treat.

[7] Voogd AC, Nielsen M, Peterse JL, Blichert-Toft M, Bartelink H, Overgaard M, van Tienhoven G, Andersen KW, Sylvester RJ, van Dongen JA (2001). Differences in risk factors for local and distant recurrence after breast-conserving therapy or mastectomy for stage I and II breast cancer: pooled results of two large European randomized trials. J Clin Oncol Off J Am Soc Clin Oncol.

[8] Soerjomataram I, Louwman MW, Ribot JG, Roukema JA, Coebergh JW (2008). An overview of prognostic factors for long-term survivors of breast cancer. Breast Cancer Res Treat.

[9] Oven Ustaalioglu BB, Balvan O, Bilici A, Develi A, Aliustaoglu M, Vardar FA, Erkol B (2015). The differences of clinicopathological factors for breast cancer in respect to time of recurrence and effect on recurrence-free survival. Clin Transl Oncol.

[10] Gur G, Rubin C, Katz M, Amit I, Citri A, Nilsson J, Amariglio N, Henriksson R, Rechavi G, Hedman H (2004). LRIG1 restricts growth factor signaling by enhancing receptor ubiquitylation and degradation. EMBO J.

[11] Laederich MB, Funes-Duran M, Yen L, Ingalla E, Wu X, Carraway KL, Sweeney C (2004). The leucine-rich repeat protein LRIG1 is a negative regulator of ErbB family receptor tyrosine kinases. J Biol Chem.

[12] Krig SR, Frietze S, Simion C, Miller JK, Fry WH, Rafidi H, Kotelawala L, Qi L, Griffith OL, Gray JW (2011). Lrig1 is an estrogen-regulated growth suppressor and correlates with longer relapse-free survival in ER alpha-positive breast cancer. Mol Cancer Res.

[13] Miller JK, Shattuck DL, Ingalla EQ, Yen L, Borowsky AD, Young LJ, Cardiff RD, Carraway KL, Sweeney C (2008). Suppression of the negative regulator LRIG1 contributes to ErbB2 overexpression in breast cancer. Cancer Res.

[14] Yokdang N, Hatakeyama J, Wald JH, Simion C, Tellez JD, Chang DZ, Swamynathan MM, Chen M, Murphy WJ, Carraway Iii KL (2016). LRIG1 opposes epithelial-to-mesenchymal transition and inhibits invasion of basal-like breast cancer cells. Oncogene..

[15] Lindquist D, Kvarnbrink S, Henriksson R, Hedman H (2014). LRIG and cancer prognosis. Acta Oncol.

[16] Thompson PA, Ljuslinder I, Tsavachidis S, Brewster A, Sahin A, Hedman H, Henriksson R, Bondy ML, Melin BS (2014). Loss of LRIG1 locus increases risk of early and late relapse of stage I/II breast cancer. Cancer Res.

[17] Ljuslinder I, Malmer B, Golovleva I, Thomasson M, Grankvist K, Hockenstrom T, Emdin S, Jonsson Y, Hedman H, Henriksson R (2005). Increased copy number at 3p14 in breast cancer. Breast Cancer Res.

[18] Ljuslinder I, Golovleva I, Henriksson R, Grankvist K, Malmer B, Hedman H (2009). Co-incidental increase in gene copy number of ERBB2 and LRIG1 in breast cancer. Breast Cancer Res.

[19] Linderholm BK, Lindh B, Beckman L, Erlanson M, Edin K, Travelin B, Bergh J, Grankvist K, Henriksson R (2003). Prognostic correlation of basic fibroblast growth factor and vascular endothelial growth factor in 1307 primary breast cancers. Clin Breast Cancer.

[20] Linderholm B, Lindh B, Tavelin B, Grankvist K, Henriksson R (2000). p53 and vascular-endothelial-growth-factor (VEGF) expression predicts outcome in 833 patients with primary breast carcinoma. Int J Cancer.

[21] Thompson PA, Brewster AM, Kim-Anh D, Baladandayuthapani V, Broom BM, Edgerton ME, Hahn KM, Murray JL, Sahin A, Tsavachidis S (2011). Selective genomic copy number imbalances and probability of recurrence in early-stage breast cancer. PLoS One.

[22] Miller KD, Siegel RL, Lin CC, Mariotto AB, Kramer JL, Rowland JH, Stein KD, Alteri R, Jemal A (2016). Cancer treatment and survivorship statistics, 2016. CA Cancer J Clin.

