# Supplementary figures and images for: LRIG1 gene copy number analysis by ddPCR and correlations to clinical factors in breast cancer
Source: BMC Cancer. 2020 May 24;20:459. doi: 10.1186/s12885-020-06919-w (PMC7245921; doi:10.1186/s12885-020-06919-w)

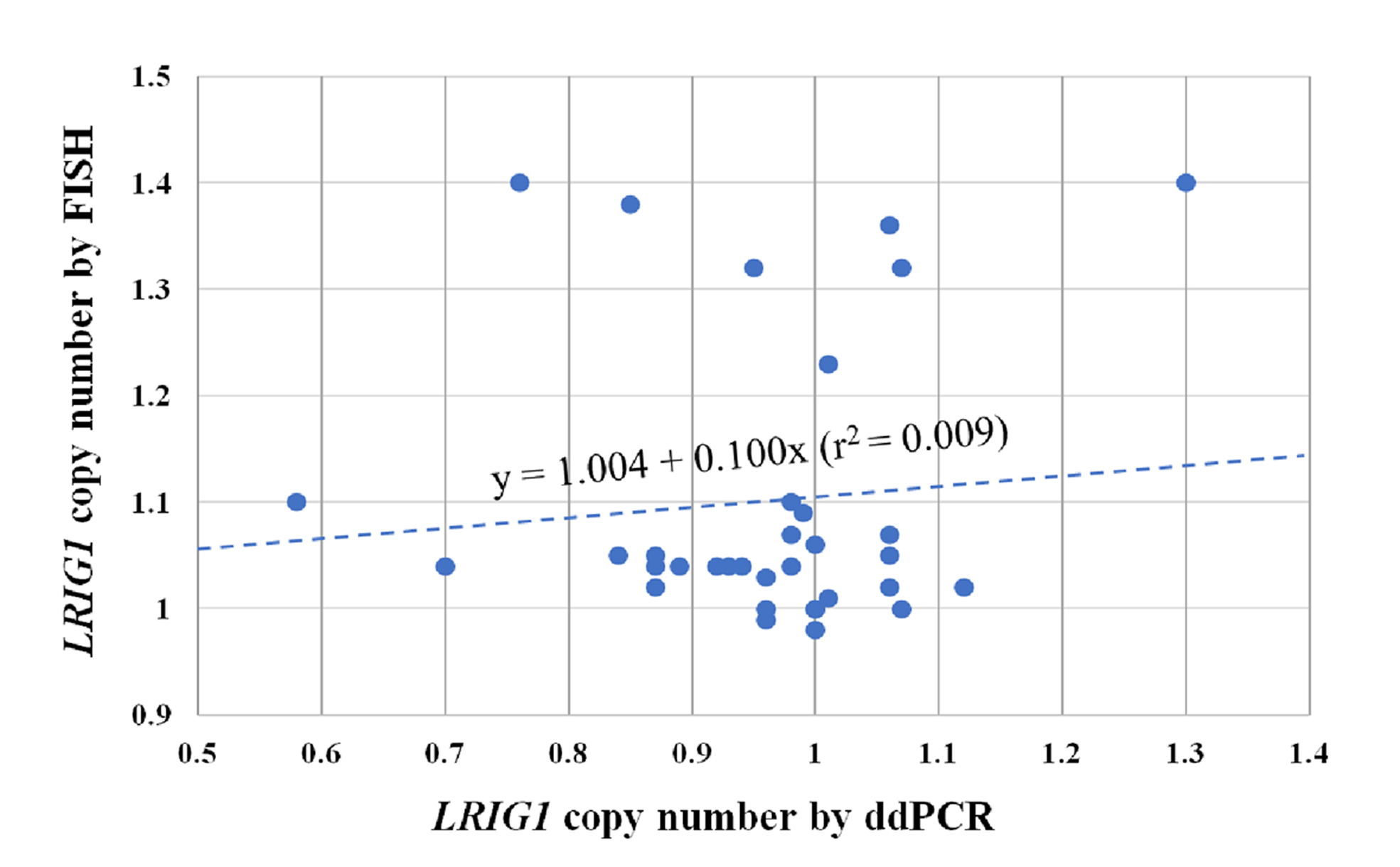

Supplement: Supplementary file 1 — Additional file 1: Fig. S1. Lack of a correlation between the ddPCR and FISH results. Dot plot showing LRIG1 copy numbers determined for 34 breast tumors using ddPCR (current study) and using FISH (Ljuslinder et al., 2009). The linear regression line (y = 1.004 + 0.100x, r2 = 0.009) is presented as a broken blue line. [file 12885_2020_6919_MOESM1_ESM.tif]

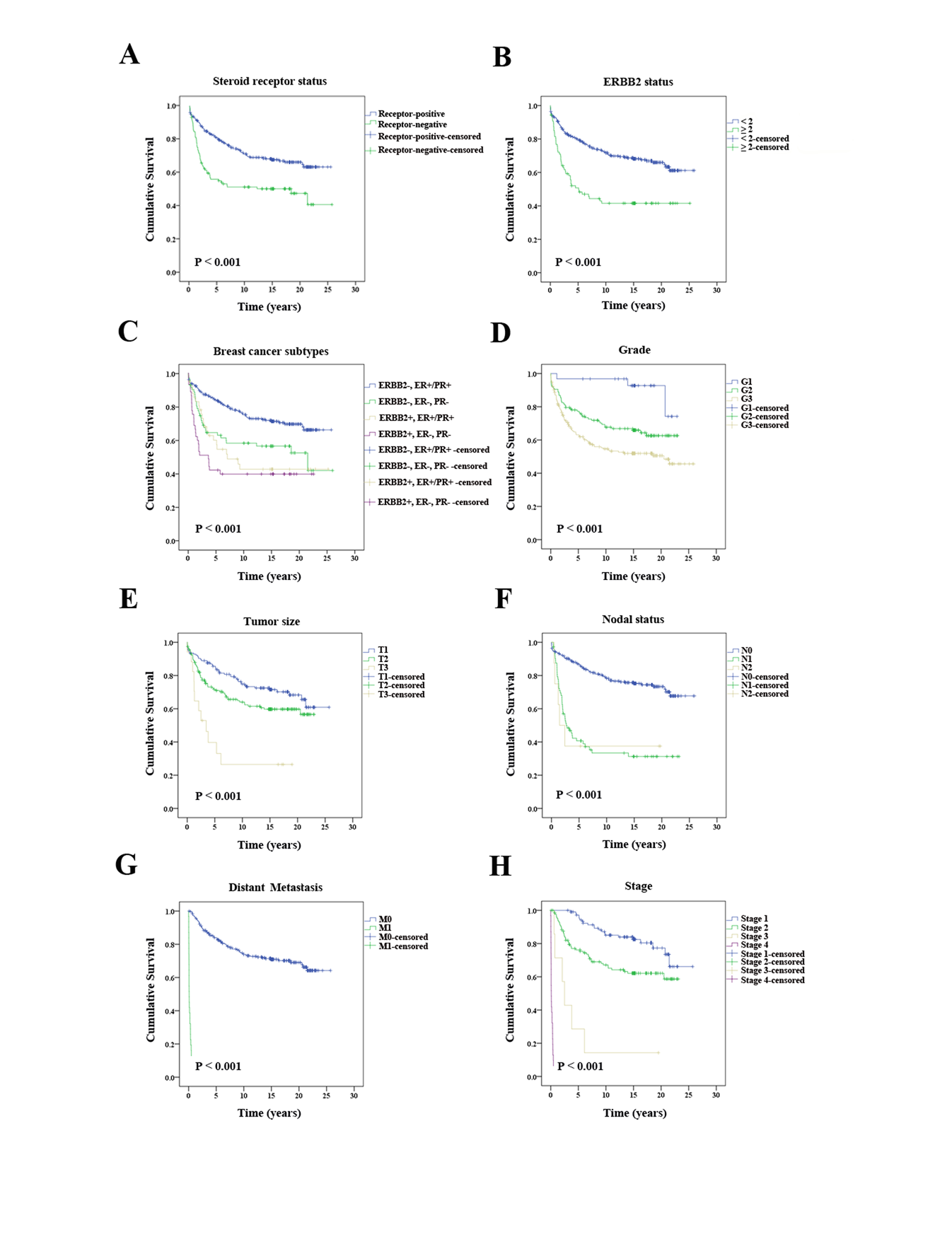

Supplement: Supplementary file 2 — Additional file 2: Fig. S2. Kaplan-Meier curves for MFS according to known risk factors. Kaplan-Meier analyses were performed for 423 breast cancer patients according to ER status (A), ERBB2 status (B), breast cancer subtype (C), tumor grade (D), tumor size (E), nodal status (F), distant metastasis (G), and disease stage (H). Statistical significance was calculated using the log-rank test and is indicated in each graph. [file 12885_2020_6919_MOESM2_ESM.tif]
