# Supplementary material for: LRIG1 gene copy number analysis by ddPCR and correlations to clinical factors in breast cancer
Source: BMC Cancer. 2020 May 24;20:459. doi: 10.1186/s12885-020-06919-w (PMC7245921; doi:10.1186/s12885-020-06919-w)
Supplement: Supplementary file 3 — Additional file 3. [file 12885_2020_6919_MOESM3_ESM.docx]

| **Gene** | **Forward primer (5'-3')** | **Reverse primer (5'>3')** | **Probe (5'>3')** | **Amplicon length (bp)** | Amplification efficiency (%) |
| --- | --- | --- | --- | --- | --- |
| *UGT1A1* | GACCCTTTCCTTCCTTGC | CCTCAAATTCCAGGCTGC | ACCTGTCTCTGCCCACTGTATTCTTC | 97 | 0.99 |
| *CHUK* | GGCATTTTAAGTAAAAGTCTACCTG | GGAAGAGAACCTGTTCAGTG | AAAGGCTGCTCACAAGTTCTATTTCTT | 113 | 0.98 |
| *NR5A1* | TGACCGTATGAGGGGTGG | TGTGCCTTCTTCTGCTGTTT | CGCTTGTACATCGGCCCAAACTTG | 81 | 0.98 |
| *HTR2C* | CTGTCAGGCAGATTCCAAGA | GCTCATTGTCACTGGCTTTC | TGCCGCCACTGCTTTGTCTG | 114 | 0.98 |
| *CYP1B1* | ATGCCATGAATTACCTGGATG | GGTCAGTTGTAATGGGAGTTC | TCCCCAACTCTTGTCACCTCGTA | 74 | 0.98 |
| *GJB2* | CCACCAACTACTACCTGTAATGA | TTCCCAAGCGACCTCTTTA | CGTTCTTTCCGATGCTGGCTACC | 108 | 0.98 |

**Supplementary tables**

**Table S1.** Reference gene ddPCR assays.

**Table S2.** *LRIG1* ddPCR assays.

| ***LRIG1* assay number** | **Forward primer (5'>3')** | **Reverse primer (5'>3')** | **Probe (5'>3')** | **Amplicon length (bp)** | Amplification efficiency (%) |
| --- | --- | --- | --- | --- | --- |
| L1-7 | GTTCGTACTCAGGGAGGG | CCAACCTGACAGTCTTCCA | CACGCACAAGCACGCACAAC | 84 | 0.95 |
| L1-8 | CTGGTAGGGTGGGTAGGA | CAAGCTAACAAGTTGGGAATGG | CTTGTGCCTGTCTCTGTATTGCCG | 108 | 0.98 |
| L1-9 | CCTGAGGAGTCACTTGAAGATAAG | GTGGGCCAGCCATCATTC | TGCCTTGTTCCTGGAAGACCTCA | 97 | 0.99 |
| L1-10 | TCGTGTGCGGTAAGACTG | ACAGGAATTACTCCCATAATGAAGA | TACCTCCTGGCTTCCTTGTCCTTC | 94 | 0.94 |
| L1-11 | TGGTGGGAGTGTAAACTGG | AGGTCATGTCGTAACTGGATG | ACAGTCTCGGCAGTTCCTCAGTT | 83 | 1.01 |
| L1-12 | TGGCTTTTCCTTTTAGGTTTAAGTC | AACCCTCATTCTACGCCTTAC | ACGGACAGATTCTACGCCTTACAGAC | 80 | 0.95 |

**Table S3.** Copy number variance of selected genes in the TCGA breast cancer data set.

| **Rank** | **Gene†** | **Chromosome** | **Number of cases with mutation*** | **Percentage of cases*** |
| --- | --- | --- | --- | --- |
| **1** | **UGT1A1** | **2q37** | **9** | **0.94** |
| **2** | **CHUK** | **10q24-q25** | **9** | **0.94** |
| 3 | ZAP70 | 2q12 | 10 | 1,0 |
| **4** | NR5A1 | **9q33** | **11** | **1.1** |
| **5** | **HTR2C** | **Xq24** | **12** | **1.2** |
| 6 | LRIG2 | 1p13.1 | 13 | 1.4 |
| **7** | **CYP1B1** | **2p22.2** | **14** | **1.5** |
| **8** | HSPD1 | 2q33.1 | 15 | 1.6 |
| **9** | **GJB2** | **13q11-q12** | **15** | **1.6** |
| 10 | GCK | 7p.15.3-p15.1 | 15 | 1.6 |
| 11 | SLC2A1 | 1p34.2 | 16 | 1.7 |
| 12 | SCN5A | 3p21 | 16 | 1.7 |
| 13 | ELN | 7q11.23 | 16 | 1.7 |
| 14 | VCP | 9p13.3 | 16 | 1.7 |
| 15 | FECH | 18q21.3 | 16 | 1.7 |
| 16 | KLK3 | 19q13.41 | 17 | 1.8 |
| 17 | LRIG3 | 12q14.1 | 18 | 1.9 |
| 18 | CASR | 3q13 | 19 | 2.0 |
| 19 | APP | 21q21.3 | 24 | 2.5 |
| 20 | POSTN | 13q13.3 | 25 | 2.6 |
| 21 | TPH2 | 12q21.1 | 28 | 2.9 |
| 22 | CACNA1A | 19p13 | 35 | 3.6 |
| 23 | POLG | 15q25 | 39 | 4.1 |

†Genes chosen as reference genes are indicated in bold.

* Case Set: Breast tumors in TCGA with sequencing and copy number data (962 samples). Data obtained via the cBio portal (<http://www.cbioportal.org/public-portal>). Dataset described at <http://www.cbioportal.org/public-portal/study.do?cancer_study_id=brca_tcga>. Data obtained via GISTIC (All GISTIC output files for TCGA are available at: gdac.broadinstitute.org)

**Table S4.** Mean *LRIG1*/reference gene ratios for different ddPCR assays among twelve healthy individuals.

| Assay pairs | Mean copy number ± SD |
| --- | --- |
| *LRIG1-7/ CYP1B1* | 0.98 ± 0.041 |
| *LRIG1-8/ UGT1A1* | 0.93 ± 0.031 |
| *LRIG1-9/ GJB2* | 1.00 ± 0.050 |
| *LRIG1-10/ HTR2C* | 0.89 ± 0.041 |
| *LRIG1-11/ CHUK* | 0.99 ± 0.029 |
| *LRIG1-12/ NR5A1* | 0.97 ± 0.030 |

**Table S5.** Clinical characteristics of breast cancer patients whose *LRIG1* copy numbers had been previously analyzed by FISH and were herein analyzed by ddPCR.

| **Number of patients** | 34 |
| --- | --- |
| **Year of diagnosis** | 1985-1995 |
| **Age at diagnosis (years, range)** | 34-79 |
| **ER receptor status (missing data = 1)** |  |
| Negative | 10 |
| Positive | 23 |
| ***ERBB2* status** |  |
| Negative | ND |
| Positive | ND |
| **Disease stage (missing data = 1)** |  |
| I | 9 |
| II | 17 |
| III | 5 |
| IV | 2 |
| **Tumor grade (missing data = 15)** |  |
| I | 0 |
| II | 8 |
| III | 11 |
| **Tumor types (missing data = 1)** |  |
| Ductal | 22 |
| Lobular | 7 |
| Others | 4 |
